# *Phytophthora* zoospores: From perception of environmental signals to inoculum formation on the host-root surface

**DOI:** 10.1016/j.csbj.2020.10.045

**Published:** 2020-11-21

**Authors:** Ilaria Bassani, Marie Larousse, Quang D. Tran, Agnès Attard, Eric Galiana

**Affiliations:** a Université Côte d'Azur, INRAE, CNRS, ISA, Sophia Antipolis 06903, France; b Université Côte d'Azur, CNRS, UMR 7010, Institut de Physique de Nice, Nice 06108, France

**Keywords:** *Phytophthora* zoospore, Motion, Perception, Soil, Microbiota, Host-root, Taxis

## Abstract

To explore moist soils and to target host plants, phytopathogenic *Phytophthora* species utilize the sensory and propulsion capabilities of the biflagellate unicellular zoospores they produce. Zoospore motion and interactions with the microenvironment are of primary importance for *Phytophthora* physiology. These are also of critical significance for plant pathology in early infection sequential events and their regulation: the directed zoospore migration toward the host, the local aggregation and adhesion at the host penetration site. In the soil, these early events preceding the root colonization are orchestrated by guidance factors, released from the soil particles in water films, or emitted within microbiota and by host plants. This signaling network is perceived by zoospores and results in coordinated behavior and preferential localization in the rhizosphere. Recent computational and structural studies suggest that rhizospheric ion and plant metabolite sensing is a key determinant in driving zoospore motion, orientation and aggregation. To reach their target, zoospores respond to various molecular, chemical and electrical stimuli. However, it is not yet clear how these signals are generated in local soil niches and which gene functions govern the sensing and subsequent responses of zoospores. Here we review studies on the soil, microbial and host-plant factors that drive zoospore motion, as well as the adaptations governing zoospore behavior. We propose several research directions that could be explored to characterize the role of zoospore microbial ecology in disease.

## 1 Introduction

Oomycetes of the genus *Phytophthora* comprise several of the most harmful plant pathogens described to date. They are responsible for serious diseases in hundreds of plant species, with massive ecological and economic losses worldwide [1], [2]. Around 120 *Phytophthora* species have been described thus far [3]. Many environmental factors have been shown to affect *Phytophthora* disease development, including climatic, chemical, physical and biological conditions that can interact with one another to induce the onset of disease [4]. At the landscape scale, moisture and wind air speed, geomorphologic and topographic features, soil clay content, and the movement of animals and humans are all traits associated with *Phytophthora* epidemiology [5], [6], [7]. This review focuses on root diseases caused by *Phytophthora* zoospores and addresses recent findings on environmental signals that lead to inoculum formation on the host surface. The emergence of disease is controlled by close proximity between roots and water flows, allowing root-to-root contact and increasing the concentration or dispersal of propagules, in addition to plant-pathogen interaction [5]. At the microenvironmental scale, the disease risk starts as soon as zoospores escape from a sporangium. Indeed, while *Phytophthora* species grow as filamentous coenocytic hyphae and produce both sexual (oospores) and asexual (sporangia, zoospores) propagules, the epidemic spread of root diseases is mainly based on dispersal in soil and water films as biflagellate zoospores [1], [8].

**Fig. 1:**
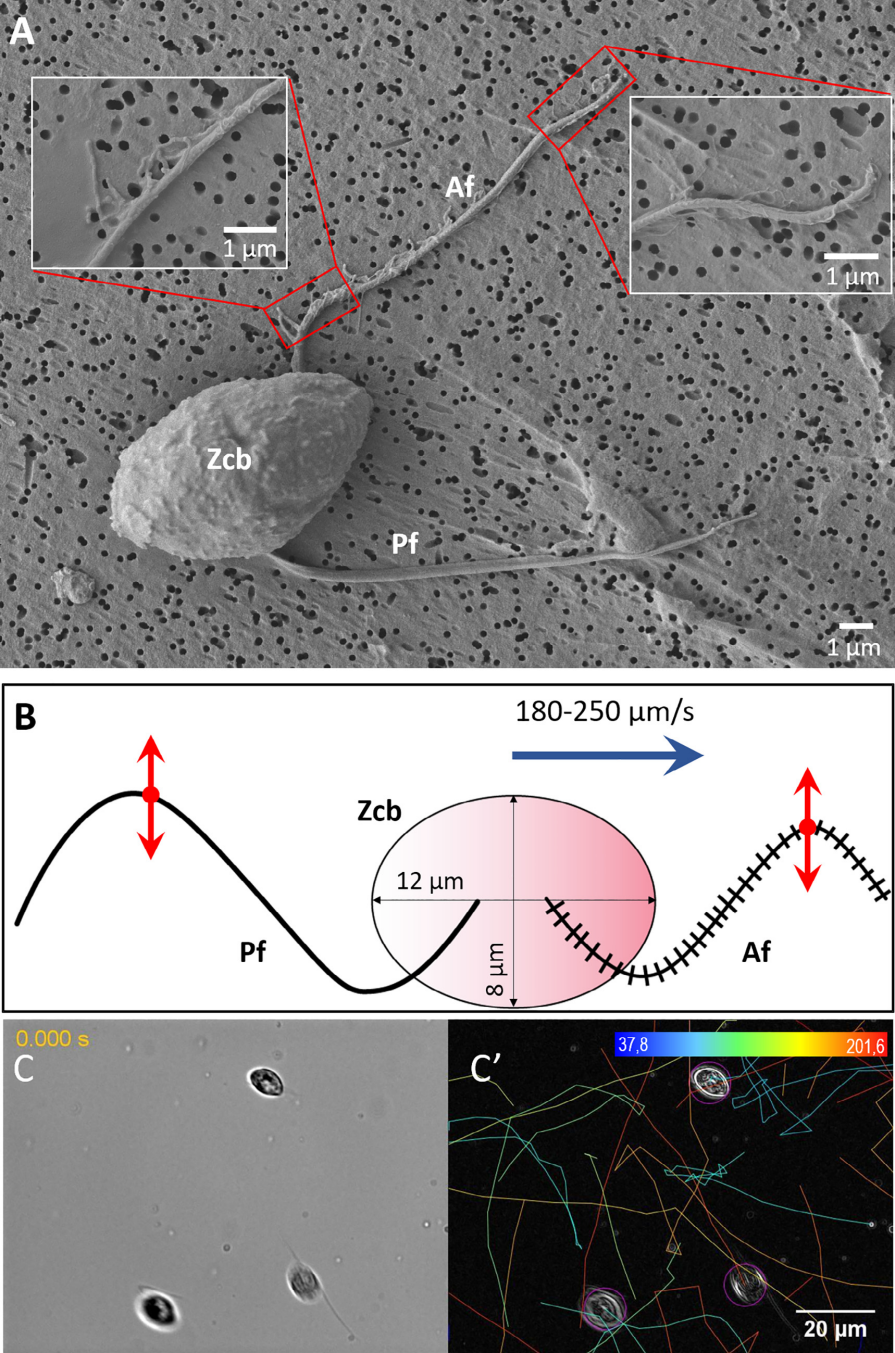
Structure and microswimmer traits of *Phytophthora* zoospores. (A) Micrograph of *P. parasitica* zoospore obtained using scanning electron microscopy (SEM). This shows the characteristic ellipsoidal zoospore cell body (Zcb) and the anterior and posterior flagella (Af and Pf, respectively). Tubular (left inset) and thinner (right inset) mastigonemes are found along the anterior flagellum, while the posterior flagellum is smooth. (B) Two-dimension schematic representation of the *P. palmivora* zoospore, including the two flagella beating with periodical waveforms in opposite directions and connected to the ellipsoidal cell body. The red arrows indicate the beating patterns of the flagella, while the blue arrow indicates the swimming direction of the zoospore. Cell body size and zoospore speed are obtained from Appiah *et al*. 2005 [9]. Panels C and C′ show *P. parasitica* zoospores swimming in water (C) and the corresponding trajectory patterns delineated using the TrackMate plugin [19] as per the procedure detailed in Galiana *et al.*[15]. The trajectories indicate the randomness in swimming speed and direction of zoospores under no constraints. (C′) Correspondence between colors and mean speed (µm/s) is indicated in the scale at the top of panel C′. (For interpretation of the references to colour in this figure legend, the reader is referred to the web version of this article.)

Zoospores are ellipsoidal, single nucleated cells that lack a cell wall. Each zoospore swims and explores randomly the environment by means of two flagella, one directing forward and the other one backward. Both flagella are inserted in a ventral groove [8] and are able to propel the cell body at high speed, up to 250 µm/s [9] (Fig. 1, Video S1 of Supplementary Data). For beating orchestration, the two flagella exhibit the same structure and repertoire of motor proteins as other eukaryotic microswimmers (such as *Chlamydomonas reinhardtii*), e.g. dyneins, which bind tubules under the control of radial spokes [1], [8], [10], [11]. The specific opposing orientation and direction of beating patterns of the flagella make *Phytophthora* zoospores a simple but attractive model to investigate the hydrodynamics of microswimmers as they explore and invade a porous medium such as the soil. When beating, the two flagella orientate the wave propagation outwards from the cell body, giving the appearance that they are competing with each other. However, the smooth, whiplash-like posterior flagellum pushes water outwards in its wave propagation, while the anterior draws the fluid toward the body thanks to multiple mastigonemes attached along the flagellum (Fig. 1) [12]. Theoretical and biological studies have established the effect of mastigonemes in reversing the thrust generated by the anterior flagellum [12], [13], with both flagella being found to generate thrust in the same direction following the wave propagation direction of the anterior flagellum. Thus, the actions of flagellar mastigonemes are critical in the determination of zoospore swimming direction, speed and propulsive efficiency.

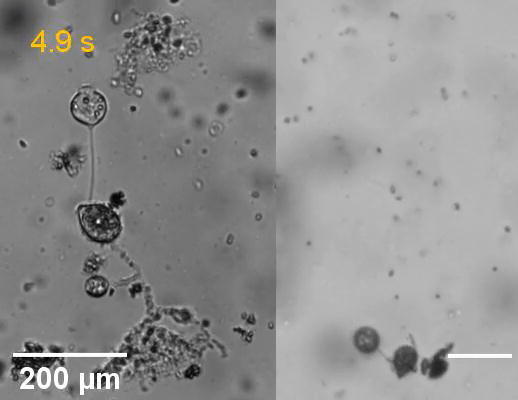

**Fig. 2:**
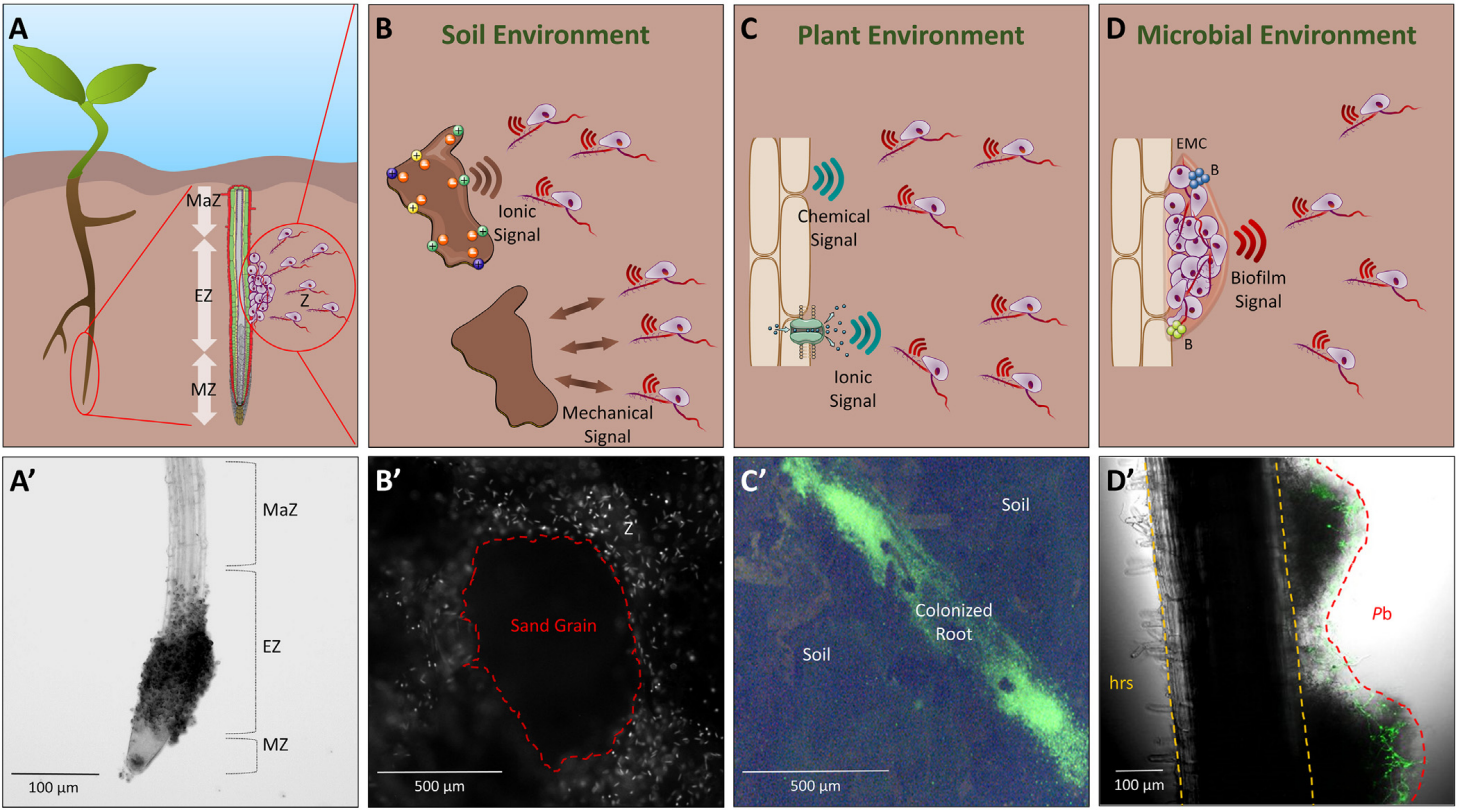
Zoospore interactions with the surrounding environment. Panel A shows a schematic representation of a plant root being colonized by zoospores (Z). The zoomed longitudinal view highlights the different zones of the root tip (maturation zone (MaZ), elongation zone (EZ) and meristematic zone (MZ)), and illustrates the preferential aggregation of zoospores at the EZ as reported by Attard et al. [20]. Panel A′ shows an EZ colonized by *P. parasitica* zoospores, 25 min after inoculation. Panels B, C and D give an overview of zoospore interactions with soil, plant and microbial environments, respectively. In Panel B, ionic signals emitted by charged soil particles and zoospore physical interactions with soil grains are represented. Panel B′ shows a fluorescence micrograph of a sand grain surrounded by zoospores (Z) that are exploring its surface. For cytoplasmic staining, zoospores were initially loaded for 10 min with 1 µM BCECF-AM (2′,7′ -bis-carboxyethyl-5(6′)-carboxyfluorescein acetoxymethyl ester). Panel C shows the ionic and chemical signals (e.g. root exudates) that are emitted or released by the plant root and subsequently attract zoospores. The fluorescence micrograph in C′ shows *P. parasitica* BCECF-stained zoospores having colonized a tomato root in the soil. The profile of fluorescence (green) illustrates the complete coverage of a tomato root by emerging *Phytophthora* mycelium (the part which can be visualized among soil elements), as the result of an extensive colonization by zoospores a 90 min soil exploration. The vast majority of zoospores had reached the root while very few were dispersed or still exploring the soil microenvironment. Panel D shows mixed biofilm formation on the root surface with incorporation of bacteria (B) and newly attracted zoospores, was well as extracellular matrix (ECM) formation. Panel D′ shows mixed biofilm formation on a tomato root surface. It illustrates the preferential colonization of the *Phytophthora* biofilm (*P*b) rather than healthy root surface (hrs), by *Pseudomonas* species expressing Green Fluorescent Protein (GFP), 2 h post bacterial inoculation [18]. (For interpretation of the references to colour in this figure legend, the reader is referred to the web version of this article.)

Our basic molecular understanding of the perception of environmental signals by zoospores has been mainly generated by *in vitro* investigations that mimic natural conditions. In the rhizosphere, the first step toward a successful infection relies on the perception of diverse stimuli at multiple levels (Fig. 2). The ion exchange dynamics between soil particles and plant roots, together with the chemical gradients generated by root exudates, dictate the direction of motion (Fig. 2B,C) and activate cell responses. This results in coordinated zoospore behavior and their preferential localization to the water film at the interface between soil particles and plant roots [8], [14], [15]. The early stages of host surface colonization involve sequentially the loss of the two flagella, the discharging of adhesive molecules and the transition to walled cysts which undergo germination before penetration and colonization [1], [8]. They may also involve zoospore population dynamics, where zoospores produce signals to attract hundreds of individuals, resulting in encystment, extracellular mucilage elaboration and biofilm formation on the plant surface [16]. Moreover, in soil, zoospores can either compete or cooperate with other rhizospheric microbiota species at the root surface (Fig. 2D) [17]. These interactions result in changes in microbiota composition [18], regulation of disease onset, as well as an additional and complex array of environmental signals that can both dictate motion direction and regulate the early steps of root surface colonization.

Soil-plant-zoospore*-*microbiota interactions are thus emerging as key events for *Phytophthora* dissemination, inoculum constitution and infection establishment. Here we review studies on environmental, microbial and host-plant factors that have been shown to drive the pre-infection behavior of zoospores before disease development. They mainly relate to (1) the displacement in the water film at the interface with soil particles; (2) the interactions with other microorganisms, (3) the early events of infection, which include rhizosphere-mediated attraction, adhesion and aggregation at the site of infection. We also draw some possible developments that would increase the understanding of the mechanisms underlying zoospores sensing and cellular responses.

## 2 The soil environment

### 2.1 Impact of soil on zoospores

As soil microswimmers, zoospores explore soil water films, air bubbles, humus, clay particles and mineral grains. They sense interfaces and surfaces, or bypass them to track their pathway toward the plant target (Fig. 2B,B′ and Video S2). Water fluxes, combined with the soil microstructure and the autopropulsion capability of zoospores, are considered as significant contributors to disease outbreaks due to their influence on zoospore distribution. Zoospores move according to the microstructure composition comprising sand, clay or loam [21]. Soil particles create repulsion/attraction force fields affecting zoospore dissemination [22] depending on the capacity of negatively charged-soil particles to hold exchangeable cations. Nutrient cations absorbed by plants are known to regulate the spatial abundances of soil bacterial communities [23]. In their dissolved form, they appear to be a pivotal element in regulating zoospore release, motion and dissemination. Ca^2+^ treatments affect zoospore release (i) during cleavage of the *P. parasitica* sporangium protoplasm into mono-nucleated cells and (ii) when zoospores are released by dissolution of the sporangium papillum [24]. *In vitro, P. cinnamomi* zoospores exhibit negative chemotaxis toward mono cations leading to collective pattern formation [25], [26]. K^+^ homeostasis influences the locomotion and the encystment of zoospores. When K^+^ is applied as a gradient, it provides guidance to *P. parasitica* zoospores and mediates aggregation [15]. These results suggest that the diffusion of cations in water films along the concentration gradient from soil particles to plant roots contributes to shape microhabitats that are favorable to *Phytophthora* zoospore dissemination and aggregate formation in the soil.

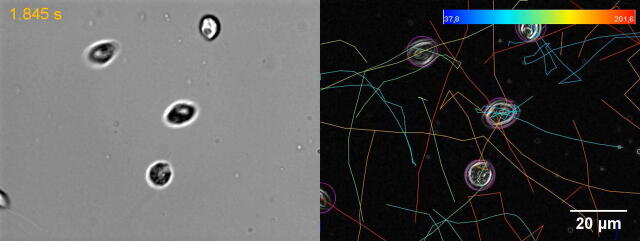

Despite these advances, little is known about the factors controlling zoospore behavior in porous media nor how these factors contribute to the zoospore’s preferential attraction to the root cues of host plants. In order to produce disease models demonstrating the incidence of a disease based on zoospore capability to reach a host as a function of soil composition, a major challenge will be the development of microfluidic devices to investigate zoospore displacement in conditions designed to mimic the nature, the geometry and the electric charges of soil particles [27], [28]. Such tools would also contribute to our understanding of how zoospores sense and respond to ion stimuli, electric fields or physical obstacles. Similar analyses could address zoospore behavior in different types of soil microstructures after having loaded zoospores with cellular probes (Fig. 2B′ and Video S2) or using *Phytophthora* strains expressing reporter genes encoding fluorescent proteins [29]. For example, an agronomic challenge will be the analysis of soils characterized by their exchange capacity of nutrient or metal cations that are used to control oomycete diseases, such as Cu^2+^ in the case of the Bordeaux Mixture. In such a case, the facilitated analysis of zoospore displacement and distribution in soil, using labeled zoospores to track them, should give information on how Cu^2+-^soil particles interactions impacting the metal retention capacity of the soil may interfere with the management of disease dissemination.

### 2.2 Impact of zoospores on soil

*Phytophthora* species may play an important role in the soil where they decompose and recycle plant materials. For instance, in a study on microbial community functional structure variations under different soil management techniques, *P. cinamomi* polygalacturonase-expressed genes were found to be among the most abundant genes related to carbon degradation [30]. We know very little about the specific roles of zoospores in ecological balance and their contribution may appear somewhat tenuous. However, the capacity of *Phytophthora* species to release 10^5^-10^6^ zoospores per infected plant in controlled conditions [16], [31] is indicative of their ability to induce radical change in the explored soil environment. For example, the 30-fold upregulation of a gene encoding a secreted alpha carbonic anhydrase (α-CA), observed in *P. parasitica* zoospores upon aggregation establishment [32], together with the oomycete’s ability to produce a high-density inoculum in proximity of the target [16], suggests a potential and transitory role of this zoospore enzyme in non-photosynthetic CO_2_ fixation. A recent study showed that soil CA activity varied with the diversity of microbial communities and CA gene expression patterns [33]. CA-mediated CO_2_ hydration capacity was assessed according to α-CA gene expression levels in algal and bacterial taxa, i.e. *Chlamydomonas reinhardtii* and Proteobacteria, even though the expression patterns were difficult to interpret due to the low representation of eukaryote metatranscriptomic data [33]. This study pointed out the lack of data on environmental CA distribution in lower eukaryotes, despite this being key to their prominent role in CO_2_ fixation. Interestingly, another study conducted on karst ecosystems revealed higher CA activity in proximity to the soil surface and plant roots with higher extracellular CA activity attributable to the fungal population, suggesting soil eukaryotic microorganisms as an important source of CA activity [34].

Expanding the *Phytophthora* representation in currently available genomic databases and developing metatranscriptomic and enzymatic activity studies on soil micro-eukaryotic communities [35], [36], [37] would contribute to the exploration of the potential role of *Phytophthora* CAs in CO_2_ fixation in the soil.

## 3 The microbial environment

### 3.1 Zoospore-zoospore interactions

An important question here is whether zoospores can sense the difference between interactions amongst themselves and interactions with the environment to eventually establish a collective motion. Collective behaviors of zoospores have been described in cell suspension, but remain poorly understood in general. Experimental evidence has demonstrated that zoospore-zoospore interactions can lead to “pattern swimming” in the absence of chemical or electrical signals. Ochiai *et al.* showed that *P. citricola* zoospores experience bioconvection pattern swimming in which the zoospores swarm to a highly concentrated spot on the fluid surface and then move downward and away from that spot due to an increase in zoospore density and the depth of fluid [38]. This suggests that zoospore-zoospore interactions are the result of zoospore response to gyrotaxis. Additionally, Savory *et al*. conducted experimental observations and proposed a mathematical model revealing that, upon bioconvection in *P. infestans* zoospores, chemotaxis leads to auto-aggregation of highly concentrated plumes, which are advantageous in attacking local targets [39]. In *P. infestans* and *P. sojae*, the silencing of the G-protein α subunit-encoding gene results in aberrant swimming patterns, characterized by a higher frequency, sharp turns and shorter-distance displacement compared to wild-type zoospores [40], [41]. Moreover, as a consequence or as a concomitant effect of aberrant motility, silencing of the same gene caused negative geotaxis (i.e. attraction toward the surface), density-dependent auto-aggregation and chemotaxis impairment *in P. infestans*, providing a preliminary indication of the molecular pathways underlying zoospore motility and collective behavior [40].

Nevertheless, these assays do not demonstrate what happens to zoospores in soil, but rather suggest density instability with zoospore motion being the determinant factor. Recent studies have shown that *P. parasitica* zoospores display collective behavior and *in vitro* aggregation patterns in response to a K^+^ external ionic gradient as a primary stimulus [13]. Aggregation is induced by a sequence of events starting with negative chemotaxis, during which zoospores move toward a region where the K^+^ concentration is < 1–4 mM, resulting in upward zoospore migration and swarming. The increased cellular density leads to bioconvection, with plume formation and downward migration, and consequent rapid aggregation [15]. Investigations at the cellular and molecular level suggested that this behavior could be regulated by cell-to-to cell signaling and cation transport because Ca^2+^ and K^+^ channels were found to be involved in K^+^ electroception and a remarkable K^+^-induced enhancement of alpha carbonic anhydrase (α-CA) activity [32].

Additionally, previous studies showed that the perception and the response to self-produced molecules determine *P. parasitica* zoospore-zoospore communication and coordinated behavior, in a way that is analogous to bacterial quorum sensing [42]. Zoospore-secreted products stimulate cyst germination and induce a tactic response to enhance zoospore auto-aggregation and infection establishment [42]. Nevertheless, the nature of these molecules and overall cellular responses that lead to coordinated behavior and aggregation remain largely uncharacterized and require further extensive characterization at both the cellular and molecular levels.

Zoospore-zoospore communication has also been proposed to occur following the attraction process to a host. *P. parasitica* appears to use such communication to amplify and increase local adhesion by forming groups of cells that occupy specific or large areas of the plant surface and undergo synchronized encystment (Fig. 2D,D′) [16]. The subsequent structure exhibits biofilm properties with mucin-like protein and polysaccharidic secretion [43], cell-to-cell adhesion, self-produced matrix formation and constitution of channels used by still-swimming zoospores for exploration [14]. The implication of the formation of such a structure on plant infection remains to be fully established. It is possible that it creates a favorable environment for the exchange of signals and/or nutrients between sessile, biofilm-associated cells and the zoospores that are still swimming [16], and/or that mucins secreted by zoospores and cysts have protective functions [44]. Another question is whether biofilm formation occurs under natural conditions, as is observed under laboratory conditions where zoospores rapidly converge at the host root surface when these cells explore porous soils (Video S3). Finally, although the molecular basis remains to be determined, it should be noted that cross-talk between zoospores of different species may occur at early stage of infection. Supernatants conditioned by zoospores of four species (*P. capsici, P. hydropathica, P. sojae*, and *P. nicotianae*) stimulate infection on different host plants (*Catharanthus roseus*, *Lupinus polyphyllus* and *Glycine* max) [45].

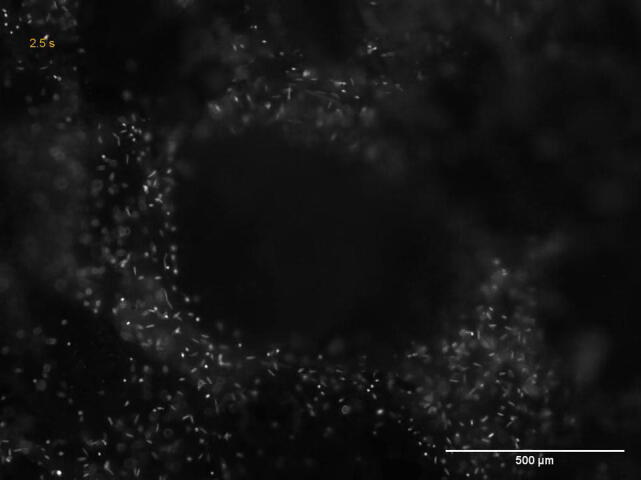

### 3.2 Zoospore interactions with other microorganisms

Zoospores explore the root environment together with the other microorganisms living in the rhizosphere. The electrical signaling mediated by bacterial ion channels that regulates the cell–cell dialogue within bacterial biofilms [46] could also drive the attraction/repulsion of neighboring zoospores through modification of the membrane potential of zoospores. This could affect the beating of the flagella or activate cellular responses, such as osmoregulation. Chemotactic signaling pathways remain to be characterized in this context.

Conversely, there is evidence showing that zoospore behavior in the rhizosphere results in physical associations with a broad range of microorganisms during root surface colonization (Fig. 2D′). This contributes to the dissemination of *Phytophthora* propagules (Video S4) [47], the inhibition of zoospore movement and hyphal growth [48] or the colonization by other microorganisms, as illustrated in Fig. 2D′ [17]. These findings, mainly descriptive, underscore the need to investigate how inoculum constitution on the host-root surface is affected by the microbial ecology of *Phytophthora*. In order to begin to delineate the *meta*-role of the microbial environment of *Phytophthora* species in the establishment of disease, studies based on 16S/18S and/or 26S rRNA sequencing have assessed microbial diversity in rhizospheric samples associated with *Phytophthora* infection [49] and compared it with that of healthy samples [18], [49]. The analyses of *Quercus* spp. and *Curcubita* microbiomes established a positive correlation between the abundance of *Trichoderma* spp. and ectomycorrhizal fungi with a lower incidence of root disease caused by *Phytophthor*a spp. [49], [50]. Investigation of the rhizospheric bacterial microbiota associated with *P. parasitica* at the root surface of *Solanum lycopersicum* demonstrated a shift in the microbial community induced by *Phytophthora* infection, involving a Bacteroidetes/Proteobacteria transition with an enrichment of sequences assigned to the Bacteroidetes phylum and a reduction in those assigned to Proteobacteria [18]. Such resources also provide a basis to define the microbial inter-kingdom interactions regulating *Phytophthora* disease outcomes, and also those caused by bacteria. For instance, opportunistic *Pseudomonas* spp. establish commensal interactions with *P. parasitica*, preferentially colonizing the oomycete rather than the roots, so that they can infect plant cells [18]. By profiling the *A. thaliana* root microbiome, Durán *et al.* (2018) provided evidence that negative interactions between bacteria and oomycetes, members of root microbiota, are critical for plant survival and maintenance of the host-microbiota balance [51]. On the other hand, the rhizospheric microbiota of some wild *Solanum* species may contribute to off-season survival and pathogenicity of *P. infestans* [52]. In the specific context of zoospore swimming in the soil, such studies would shed light on how zoospores maximize microbial interactions during the early infection events to exploit the diversity of the effector repertoire that each species uses to promote infection [53], [54]. The study of *Phytophthora* microbiota is also relevant to addressing the challenges associated with reducing pesticide use and developing bio-based materials for biocontrol and diagnostics [55].

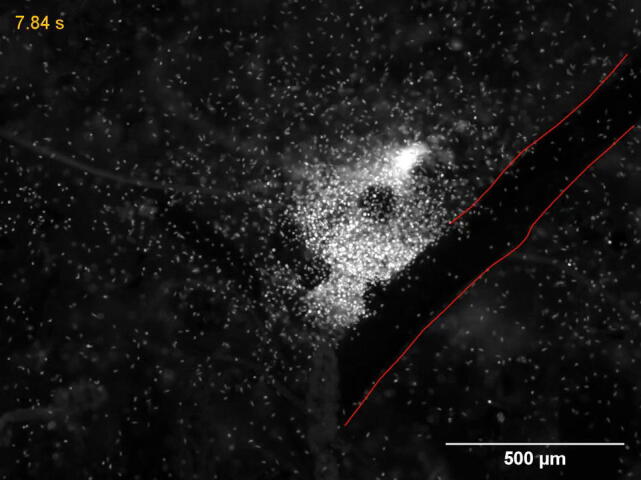

## 4 The plant environment: The rhizosphere

The rhizosphere is the dynamic and heterogeneous soil space around the roots that is characterized by various connections between solid, liquid and gaseous substances and living species [36]. It has a pivotal role in plant growth promotion and nutrition [56]. The composition of the rhizosphere is mainly influenced by root soil acidification, H^+^ exchange, nutrient uptake and release of a wide range of exudates (sugars, polysaccharides, organic acids, sterols, phenolics, proteins, secondary metabolites and ions). Each of these compounds form a gradient across the rhizosphere along the longitudinal axis of the root. They are involved in attracting or repulsing beneficial and pathogenic microorganisms (Fig. 2C) [57], [58], [59]. Investigations addressing how and which rhizospheric compounds direct zoospore chemotaxis toward roots have resulted in the identification of stimuli among root exudates. For instance, *P. palmivora* is attracted by isovaleraldehyde, valeraldehyde and ante-isovaleraldehyde, compounds present in root exudates of many plants; *P. sojae* is attracted by isoflavones (daidzein, genistein) secreted by soybean roots [60]; and *Phytophthora* spp*.* are attracted by amino acids (aspartate, glutamate, asparagine, glutamine, arginine, methionine) secreted by many plants [8]. Ethanol is secreted by flooded roots and also attracts zoospores [60]. Despite molecular patterns governing chemotactic response remaining largely unknown, previous studies have reported the involvement of the G-protein signaling pathway in response to chemoattractants such as aspartate and glutamate in *P. infestans* [40], and daidzein and soybean roots in *P. sojae* [41], [61], [62].

Other studies have shown that *Phytophthora* zoospore motion is also driven by electric fields (electrotaxis) that are differentially produced by roots along their axes (Fig. 2C). *P. palmivora* presents an anodic taxis that drives zoospores to the rye grass root elongation zone (EZ) [14]. In different host species [14], [20], [63], zoospores preferentially aggregate at the root EZ (Fig. 2A,A′) prior to penetration [20]. The EZ is the initial site of root cell growth where a shift to high rates of proton efflux generally begins and is controlled by the activity of plasma membrane proton pumps. This proton gradient contributes to the turgor pressure required to drive cell expansion and facilitate mineral nutrient uptake (e.g., K^+^, Na^+^, Ca^2+^, Mg^2+^, Cl^-^; [64]). Ionic exchanges and surface generated gradients associated with root growth in the EZ may play a crucial role in plant-*Phytophthora* interaction.

Thus, a number of root attractants and repellents for zoospores have been characterized. It is now important to define the parameters of effective chemoattraction at root surfaces. This will require characterization of the conditions that are necessary for the establishment of a stable gradient and chemoattraction. In this context, several parameters have to be defined: the spatio-temporal and concentration-scale of gradients at the root surface, the layer near the root in which gradients are stable [65], the zoospore distribution and the metrics of zoospore motion (velocity and trajectory) [15] in this environment. It is equally important to determine the genetic basis governing the release of attractants by the host plant. In particular, mutant screening strategies should be used to characterize the molecular actors (ions channels, transporters) and cellular mechanisms (secretory pathways, osmoregulation, nutrition of root cells, cell differentiation) regulating zoospore attraction. These studies will accelerate knowledge in the field of plant pathology, which has been somewhat overlooked in recent years, and could yield promising new targets for molecular breeding.

## 5 Concluding remarks

The versatile adaptive ability of zoospores to sense their environment is one of the key features of their evolutionary success in targeting host tissue. The availability of gene sequences and mRNA-level quantification data generated by the different *Phytophthora* genome projects has had a massive impact on the definition and the classification of the molecular repertoire at different stages of the *Phytophthora* life cycle, including zoospores [2]. Several studies have indicated the occurrence and the importance of putative pumps, ligand-gated channels, tyrosine kinase-like and G-protein signaling [32], [66], [67] in *Phytophthora*. Nevertheless, to date, very little is known about the major classes of receptor and signaling pathways involved in environment perception. The few available studies pointed out the role of a novel class of G-protein-coupled receptors (GPCRs) [61] and of the G-protein α subunit and its interacting protein, PsHint1 [41], [62] in chemotaxis toward isoflavones and soybean roots, similarly to that observed for aspartate and glutamate in *P. infestans* [40]. To further advance this field, the next step will require a shift of focus toward the genetics and biochemistry of directional taxis mechanisms on both sides: the zoospore and plant cell.

The anterior flagellum is covered with mastigonemes and may constitute a nodal point to couple the chemical, electro- or mechanosensory pathways with the reconfiguration of the motor apparatus during different phases of motion. The asymmetric position of the two flagella with regard to the perception of a stimulus during forward motion, and/or the variation in their plasma membrane components could underpin the differential responses of each flagellum to each kind of taxis [68]. To describe these mechanisms, the metrics of flagella beating need to be carefully examined by high-speed camera analyses in microfluidic environments to mimic soil and plant surface compositions. The application of microfluidics on zoospore research has not yet been fully exploited, but is undoubtedly becoming indispensable.

A mathematical model is also needed to fully understand the fundamentals behind the straight swimming trajectories, change of direction, and zoospore-zoospore and zoospore-obstacle interactions. Modeling methods of eukaryotic swimmers at low Reynolds numbers usually require a solution for the Stokes equation applied to flagellar motion to calculate the resultant cell body movement [69]. Resistive force theory and slender-body theory are the modeling methods most frequently used to predict forces and movement. In the case of zoospores, the hydrodynamics of an individual can be established by a simple microswimmer model consisting of an ellipsoidal body and two flagella beating in periodical waveforms in opposite directions, to quantitatively characterize the activity of the two flagella and the propulsive efficiency they produce. Moreover, a novel approach in quantifying the characteristics of microswimmers is to exploit the universal distributions of their specific dynamical properties that are a consequence of the variety of swimmer morphologies and sizes [70]. Thus, the zoospore hydrodynamic model is important, as we can derive the universal swimming speed of zoospores from the characteristics of their flagella and bodies.

Further advancement of the research summarized here will provide information on the behavior of zoospores in the wild, and the importance of soil reservoirs and environmental factors for survival, exploration, inoculum density and, finally, disease transmission. This will also deepen our understanding of the epidemiological processes by which the abiotic and biotic environment affects plants infection by *Phytophthora* species and the subsequent disease development.

## CRediT authorship contribution statement

**Ilaria Bassani:** Writing - original draft, Visualization, Funding acquisition. **Marie Larousse:** Writing - original draft, Visualization. **Quang D. Tran:** Writing - original draft, Visualization. **Agnès Attard:** Writing - original draft, Visualization, Funding acquisition, Project administration. **Eric Galiana:** Writing - original draft, Visualization, Funding acquisition, Project administration.

## Declaration of Competing Interest

The authors declare that they have no known competing financial interests or personal relationships that could have appeared to influence the work reported in this paper.
